# Incontinence of Urine in Children

**Published:** 1850-11

**Authors:** 


*Incontinence of Urine in Children.* By J. Simon, Esq., F. R. S.

Irritability of the bladder in children usually takes, with more or less completeness, the form of *incontinence of urine:* the child wets its bed. Whenever this symptom is presented to you, if you proceed to examine the urine, (as in every such case you should do,) you may pretty confidently expect to find copious crystals of lithic acid. This condition of the urine in children is very far from painless; and in severe cases the symptoms cannot at first sight be distinguished from those of calculate. The child makes water very often, and a little at a time, doubles itself up, and cries with the pain of each effort, and pinches and pulls its prepuce, just as it would with stone in the bladder. The pain experienced is a severe scalding in the urethra, and sometimes this passage will be so much irritated as to inflame and secrete pus. There was recently a case under my treatment which, though not one of incontinence of urine (for it was in an adult,) will yet serve to show the manner of dealing with such inconveniences, generally as depend on the passage of crystals of lithic acid in the urine. The patient, W. M., aged 22, had for two or three years suffered occasionally with symptoms, which made it probable that he had a calculus lodged in his left kidney; but the immediate cause of his admission to the hospital was the circumstance of his then habitually passing lithic acid gravel, occasionally mixed with blood. His urination was frequent and painful; his pulse was feeble, and he was of little muscular power; his skin acted fairly; his tongue was white and coated; his bowels a little constipated. I ordered him five grains of Plummer's pill every night till his tongue was quite clean, and then changed the treatment; giving him quin. disulph. gr. ii. twice a day, and potass. bicarbon. half a drachm, five hours after his chief meal. He left the hospital after a month's stay, quite free from uneasiness in his urinary organs, and materially improved in general health.

This case will illustrate the sort of treatment which I generally pursue in similar instances of chemical derangement of the urine. If the tongue is coated, and if (as is usually the case with children) the intestinal secretions are unhealthy, I give hydrarg. c. creta, or some other preparation of mercury, till that evil is remedied; I then commence the exhibition of alkalies, giving usually a single large dose daily, after the completion of the digestion of the chief meal of the day; and almost invariably I find it highly advantageous to give quinine twice a day during the same period. In my hands it has answered far better than any preparation of iron, and especially so in the combination I have mentioned. I give it usually before breakfast and before dinner, and the alkali, in copious solution, five hours after the latter meal. Extreme attention to the quantity, quality, and simplicity of the diet, is essential.

With this treatment you will seldom, I think, have occasion to resort to blistering over the sacrum, and other measures of a similar nature, which have been recommended for the cure of incontinence of urine in children. —*Lancet.*

